# Atlanta Residents’ Knowledge Regarding Heavy Metal Exposures and Remediation in Urban Agriculture

**DOI:** 10.3390/ijerph17062069

**Published:** 2020-03-20

**Authors:** Lauren Balotin, Samantha Distler, Antoinette Williams, Samuel J.W. Peters, Candis M. Hunter, Chris Theal, Gil Frank, Taranji Alvarado, Rosario Hernandez, Arthur Hines, Eri Saikawa

**Affiliations:** 1 Department of Environmental Sciences, Emory University, 400 Dowman Drive, Atlanta, GA 30322, USA; 2 Department of Health Policy, Emory University, 1518 Clifton Rd. Atlanta, GA 30322, USA; 3 Department of Environmental Health, Emory University, 1518 Clifton Rd. Atlanta, GA 30322; USA; windsor.peters@outlook.com (S.J.W.P.);; 4 Historic Westside Gardens Atlanta, Inc., Atlanta, GA 30314, USAwestsidegrowersatl@gmail.com (R.H.);

**Keywords:** heavy metals, gardening, Atlanta, soil, lead, arsenic, urban agriculture

## Abstract

Urban agriculture and gardening provide many health benefits, but the soil is sometimes at risk of heavy metal and metalloid (HMM) contamination. HMM, such as lead and arsenic, can result in adverse health effects for humans. Gardeners may face exposure to these contaminants because of their regular contact with soil and consumption of produce grown in urban areas. However, there is a lack of research regarding whether differential exposure to HMM may be attributed to differential knowledge of exposure sources. In 2018, industrial slag and hazardous levels of soil contamination were detected in West Atlanta. We conducted community-engaged research through surveys and follow-up interviews to understand awareness of slag, HMM in soil, and potential remediation options. Home gardeners were more likely to recognize HMM health effects and to cite health as a significant benefit of gardening than community gardeners. In terms of knowledge, participants were concerned about the potential health effects of contaminants in soil yet unconcerned with produce in their gardens. Gardeners’ knowledge on sources of HMM exposure and methods for remediation were low and varied based on racial group.

## 1. Introduction

The rise of urban agriculture through municipal farms and gardens has provided a strategy to cope with the food needs of a rapidly growing population [1,2]. In 2014, 54% of the world’s population lived in urban areas, and this proportion is projected to increase to 66% by 2050 [3]. In 2008, roughly one million households in the United States were involved with some community gardening efforts [4]. The use of local garden-grown food has also improved cost-savings by reducing the need to buy produce at grocery stores [5,6]. Previous community-engaged studies have cited an increase in the consumption of fruits and vegetables and a decrease in the consumption of packaged foods among individuals with access to an urban garden [5,7,8,9,10]. Shared gardens also provide an outlet for community members to engage with each other regularly as they share tools, cultures, and ideas. Previous studies have found that community gardeners often work collaboratively with each other and frequently share gardening tips and ideas among their network [5,11]. 

However, because of the nature of cities, urban gardens are more likely than suburban and rural gardens to be near areas with high pollution, such as industrial sites and roads [12]. Municipal soils typically have higher levels of heavy metal and metalloid (HMM) contaminants than non-municipal soils due to human activity, such as fossil fuel use, waste incineration, construction with lead-based paints, and mining and smelting [13,14]. Lead, cadmium, mercury, and arsenic are four HMM often associated with soil contamination [15]. Gardening increases individual exposure to these HMM through finger-to-mouth contact in children who have been playing in contaminated soils [16,17]. Gardeners may also consume the produce grown in contaminated soils or track it into their house when they do not thoroughly wash their hands or clean their shoes [4]. Ramirez-Andreotta et al. (2013) [18] found home gardeners near mining sites were susceptible to lead contamination in their food and recommended that they test their soil and modify their behaviors to limit soil ingestion. 

Lead has severe health consequences, especially for neurobehavioral-cognitive performance, such as irritability, memory loss, antisocial behavior, and reduced intelligence quotient (IQ), and attention span [19,20,21]. Exposure to arsenic is linked to increased risk of cancer, neurological and behavioral conditions, diabetes, and hearing loss [22,23]. Despite the health risks associated with HMM, community awareness of soil contaminant risks and remediation sources is limited, partially due to lack of educational resources [19]. Kim et al. (2014) [12] found that while most gardeners in Baltimore, Maryland were aware of lead as a soil contaminant, they were less aware of other HMM, such as arsenic and cadmium. In another interview-based study by Johnson et al. (2016) [24], fewer than 50% of gardeners in predominantly African American and Latino communities were aware of and concerned about the potential health effects of lead in their soil. 

Further exacerbating the issue, HMM can generally remain in soils indefinitely without degrading, and remediation techniques are often costly and difficult to carry out [25]. Therefore, contaminated soils and crops must be treated and managed to remove HMM and reduce the risk of negative health effects for gardeners. For instance, through phytoremediation, certain plants grown in contaminated soils can extract and remove HMM from the soil [25,26]. Other remedial management options include surface capping, encapsulation, electrokinetic extraction, soil flushing, chemical immobilization, bioremediation, and soil washing [27]. These techniques employ both in-situ strategies, which treat soil on-site and are usually cheaper options, or ex-situ strategies, which require contaminated soil to be removed from its original site and transported to secure treatment facilities but at a higher cost [27]. Remediation can take place through physical, chemical, electrical, thermal, or biological treatment options [27]. Previous research from Tacoma, Seattle, and Kansas City found that urban gardeners had minimal confidence in their knowledge of and proper access to resources for soil contamination [28]. Moreover, soil testing for urban gardeners is limited, and many urban gardeners lack appropriate knowledge regarding the methods for finding a soil contaminant testing lab [19]. Without awareness of soil contamination risks, community gardeners may be unlikely to seek soil remediation. 

Differences in exposure to environmental harms and unequal distribution of resources are two factors that influence the need for environmental justice. These differences may be due to variations in socioeconomic status, gender, race and/or ethnicity, age, and family structure [29]. Differences in these areas may affect one’s ability to cope with environmental hazards, including hazards of soil contaminants in the built environment and improper industrial waste disposal in low-income communities. In many cities, HMM contaminants and industrial hazards have also afflicted certain racial groups disproportionately [30]. For instance, predominantly low-income African American communities in Chicago, Illinois, and Flint, Michigan, have experienced exceptionally high levels of childhood lead poisoning [31,32]. Improved food security and nutrition also reduce environmental health inequalities and promote environmental justice by providing a secure source of healthy produce to the often disadvantaged communities [33,34]. 

The census shows that poverty has been substantially increasing in Atlanta [35]. Additionally, neighborhoods in Atlanta are vulnerable to sources of HMM contamination. Deocampo et al. (2012) [36] suggests that emissions near roads have resulted in high levels of lead in road dust. Atlanta has also historically been home to industrial sites, such as the Atlantic Steel Mill. 

Peters (2019) [37] sampled sites in West Atlanta to measure baseline HMM soil contamination in the area. Of these sites, several were found to have lead contamination that exceeded the Environmental Protection Agency (EPA) residential screening limits. Slag, a metal waste scrap created during the smelting or refining of metal ore, was also discovered in August of 2018 in West Atlanta. Slag can leach HMM contaminants into soil and plants, which can then be transferred to animals and humans who consume these plants [38]. Large quantities of slag are especially likely to be dumped in areas of the U.S. where iron and steel mills have historically been located [39]. 

Given the detection of slag dumpsites in the neighborhood, this study sought to understand the knowledge of potentially vulnerable residents better. Additional research in this area could help determine whether different levels of knowledge are associated with different levels of exposure. Through surveys and follow-up interviews, our study was conducted to explore whether Atlanta community and home gardeners were aware of soil contaminant sources and potential remediation options. Special consideration was given to using the research as an outlet for educating community members on HMM exposure, dietary-related personal health, and soil health (the capacity of soil to sustain plants, animals, and other components of the ecosystem). In the past, many researchers have not reported information or educational materials to study participants to promote environmental health literacy [40]. However, such studies provide an opportunity for researchers to educate community members about environmental health risks and potential opportunities for improvement. This study sought to integrate educational opportunities to provide community members and urban gardeners with these benefits. 

## 2. Materials and Methods 

Surveys were collected from January 2019 to March 2019 throughout the Atlanta area, with a focus on gardeners associated with Historic Westside Gardens Atlanta, Inc. (HWG). HWG is an organization dedicated to providing individuals in West Atlanta with resources and education necessary to develop and maintain community and home gardens. The anonymous survey had 37 questions and asked a combination of general demographic and personal knowledge/awareness questions. The survey took 10–20 minutes for each study participant to complete. There was a combination of multiple-choice and open-ended/short answer questions. Demographic questions asked about age, race, number of children in the household, and average annual household income. At the end of the survey, participants were given the option to provide their contact information for follow-up, individual interviews. Each interview was recorded with the permission of the interviewees, and all interviews were transcribed for analytical purposes. Interviews were conducted either in person or by phone, and nine interviews were conducted in total. Quotes from interviews were used as supplements to surveys to support and/or refute findings from survey data. 

Both paper surveys and online surveys were offered to gardeners to increase the response rate for potential participants who did not have access to a computer and/or internet. Study participants and gardening organizations were contacted through a variety of methods to increase the diversity of potential participants. The American Community Gardening Association’s database of community gardens in Atlanta was used to recruit many of the community gardeners who participated in the study. We contacted 48 community gardening organizations to reach a variety of gardeners and obtain an adequate number of responses. Surveys were also administered at weekly HWG meetings and during an Atlanta Science Festival (ASF) event at an HWG growing site. To increase survey response rates, we sent gardeners and gardening organizations follow-up reminders requesting participation and informed them of how their feedback would be used. 

Inclusion criteria for participants were to be at least 18 years old, residents of Atlanta, and actively engaged in either a community garden and/or a home garden. Each participant signed written consent forms before the study was administered. Participants were informed that their names would not be directly associated with any quotes included in the study. Community researchers and representatives from HWG who help lead community and residential gardening efforts were included in the development of research questions. 

Participants were asked to describe prior knowledge of HMM soil contaminants and their health effects, as well as whether they felt that they had adequate resources to learn more about the contaminants. When developing outline questions for the interviews, special consideration was given to previous research and best practices regarding the use of social science methods for environmental health studies [41,42,43,44]. A full list of the survey questions can be found in Appendix A. 

Participants were not offered direct compensation for engaging in the study. However, they were encouraged to collect soil from their yards and gardens to bring to the ASF event for free HMM contamination testing. Participants were also offered help with collecting these soil samples and transporting them to the ASF event for testing to make the process and survey more accessible to as many participants as possible. 

Based on past studies and knowledge of common gardening practices in Atlanta, we hypothesized that individuals participating in community gardens would be more aware of the health effects of heavy metal soil contaminants and possible remediation strategies than individuals participating only in private home gardens. This hypothesis was made under the assumption that community gardens may provide more opportunities for education regarding gardening and urban agriculture. 

### 2.1. Social Science, Community Education, and Outreach 

Social science methods, such as surveys and interviews, provide forums for researchers to gather a variety of thoughts and opinions to understand how a community feels and behaves as a whole. For instance, individual interviews are beneficial because researchers can ask more specific questions about personal matters, such as health or income [41]. Lobdell et al. (2005) [43] suggests that social science can play a key role in environmental health studies, especially studies that seek to identify risk perceptions of environmental exposures and develop awareness for limiting exposure to harmful environmental contaminants. 

Given the outreach-based objectives of this study, each survey concluded with details on attending the ASF event. Participants recruited at the ASF event were provided with additional information about the opportunities at ASF for education and outreach related to HMM contamination. Each participant was given a flyer for soil testing at ASF, and each participant was offered educational handouts provided by the Centers for Disease Control and Prevention, the Agency for Toxic Substances and Disease Registry, and the EPA. Handouts provided information on safe practices for gardening in HMM contaminated soils, effective methods to protect oneself and one’s children from lead poisoning, and ways to recognize lead poisoning. All participants were provided with contact information for researchers so they could ask follow-up questions. Various resources were also posted on the project website at https://atlsoilsafety.com. 

The study concluded with an outreach event at a final ASF event, entitled “Getting Dirty: Exploring Soil on Atlanta Farms” on May 9th, 2019. The event included an urban farm tour focused on soil contamination and remediation education, as well as a booth where community soil samples were analyzed with x-ray fluorescence. All participants received seeds and a garden startup kit for attending the event. 

### 2.2. Coding and Statistics 

A codebook was developed in the programming language R (Version 3.5.1, R Foundation for Statistical Computing, Vienna, Austria) based on the survey questions and patterns from the initial responses [45]. Questions with multiple-choice answers were combined into one categorical variable for analysis rather than multiple binary variables. Results were compiled to a complete data set including questions, question numbers, and all responses in a readable format. Upon review of final survey responses, the coding schema was adjusted accordingly. 

Survey responses for categorical questions were sorted into categories by two researchers. The two researchers compared their groupings and ensured that groupings matched before finalizing them. For questions asking whether participants were or were not aware of health effects/sources of exposure to HMM soil contaminants, follow-up questions were included to ensure that participants who answered “yes” were correct in their knowledge (Q22/Q23, Q24/Q25, and Q27/28 in Appendix A). Researchers checked these follow-up answers to ensure that participants who indicated “yes” but incorrectly described health effects/sources of exposure were not counted as aware and were grouped with those who originally answered “no” instead. Fisher’s exact test was used to analyze qualitative/categorical data statistically. 

Follow-up interview quotes best-representing themes from the survey results are also included in the results section. These quotes exemplify the main patterns detected through coding the survey responses. 

### 2.3. Ethical Considerations 

All study protocol, interview/focus group questions, and flyers were reviewed and subsequently approved by Emory University’s Institutional Review Board (IRB). All participants were informed that they were not required to answer any survey questions that they did not wish to answer and had the right to withdraw at any time. Participants were also informed that their names would not appear in any report or publication from this project. 

## 3. Results

### 3.1. Demographics 

Fifty-one gardeners from across metro-Atlanta responded to the survey, and nine interviews were conducted among these respondents. Nine of the 48 community gardening organizations contacted through the American Community Gardening Association’s database responded to the survey. Through this database, 21 respondents were recruited. Respondents from the HWG meetings were a combination of both community and home gardeners, and respondents from ASF were primarily home gardeners. Eight survey respondents were gardeners through HWG, and 26 survey respondents were recruited through the ASF event. Four respondents were recruited from more than one method. Ten respondents did not complete the entire survey due to time and interest constraints. Given that gardening organizations were partially responsible for disseminating the survey to local gardeners, it was not possible to determine how many survey respondents were contacted.

Of the survey respondents, 61% were white/Caucasian, and 26% were black/African American. Other respondents were Hispanic or Latino, Asian or Asian American, or American Indian or Alaskan Native. Sixty-eight percent of respondents identified as female and the median age group of participants was 35–44 years old. Thirty-seven percent had an average annual household income of less than $50,000, 28.3% had an average annual household income of $50,000–89,999, and 35% had an average annual household income of $90,000 or more. Twenty-two percent of respondents participated in both home and community gardens. Twenty-three percent of respondents participated in only community gardens, and 45% participated in only home gardens. Additional demographic data can be found in Appendix B. 

### 3.2. Perceived Benefits of Gardening 

**Figure 1:**
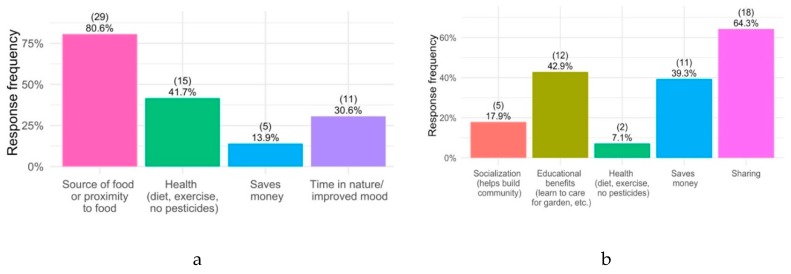
Most common perceived benefits of home gardeners (**a**) and community gardeners (**b**).

Respondents described four main benefits of participating in a home garden: proximity/ease of access to food, improved health (in terms of diet, exercise, and lack of pesticides), cost savings, and time spent in nature/improved mood (Figure 1). Respondents most frequently described proximity/ease of access to food, with 81% of respondents mentioning it as a benefit. Additionally, 42% of participants cited improved health. Almost all respondents to the survey (93%) said that they did eat food grown in their gardens. Gardeners also described that they felt “in tune with nature” when engaging in home gardening and found gardening to be “therapeutic” and helpful in promoting “self-sufficiency.” 

Respondents who engaged in community gardening activities described five main categories of benefits: Community-building/socialization, education (i.e., on proper gardening techniques), improved health (in terms of diet, exercise, and lack of pesticides), cost savings, and sharing of tools and knowledge (Figure 1). Sharing was the most frequently described benefit of community gardening, and it was listed among 64% of respondents who said they participated in a community garden. Participants described an improvement in their ability to share tools, land, infrastructure, and knowledge with others. As one participant described in a follow-up interview:

[Without a community garden], I just wouldn’t have as much space. I live in a townhouse community, so I don’t have my own yard. The community garden gives me space to garden that I wouldn’t have access to otherwise… Also, just being able to interact with other gardeners, people who are interested in gardening and growing food the same as I am is nice. I appreciate that… I also like being able to help others access an ability to grow their own food.

Educational benefits and cost savings were also frequently described among community gardeners, although sources of and proximity to food were not. Of all respondents who participated in community gardens, 43% expressed satisfaction with the opportunity to educate themselves and their children on healthy gardening techniques, while 39% suggested that cost savings were a significant reason for their engagement with community gardens. Health was much less frequently cited as a benefit for community gardeners than home gardeners. About 7% of community gardeners said health was an important benefit of engagement, while 41% of home gardeners said so. 

### 3.3. Knowledge and Concern for HMM Soil Contaminants 

**Figure 2:**
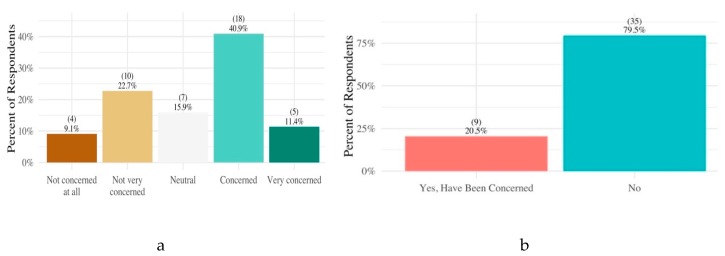
Level of concern of participants with potential health effects of hmm contaminants (**a**) and produce grown in their gardens (**b**).

More than half of survey participants indicated that they were concerned about the potential health effects of HMM contaminants in soil, yet most were generally unconcerned with eating and/or working with produce in their gardens even if their soil had never been tested for contaminants before. Of all respondents to the survey, 11% said that they were “very concerned” about the health effects of HMM in soil, and 41% said that they were “concerned.” In contrast, 32% said that they were “not concerned” or “not concerned at all” with these possible health effects (Figure 2a). Despite the high percentage who said they were concerned about potential health effects, 80% of all participants indicated that they did not have any concerns for themselves or their families about eating and working with produce grown in their gardens (Figure 2b).

There was a difference in participants’ perspectives on health based on race and the types of gardens too. Forty-five percent of black/African American respondents were “concerned” or “very concerned” about the health effects of HMM in soil, in comparison with 53.1% of white/Caucasian respondents (Table 1). Twenty-seven percent of black/African American respondents were “not concerned at all” or “not very concerned,” in comparison to 40% of white/Caucasian respondents. Individuals involved with a home garden were also more likely to be worried about HMM contaminants than those involved with a community garden. Of all participants involved with a home garden, 55% were “concerned” or “very concerned” about HMM contaminants in comparison to 40% of those involved with a community garden (Table 1). Participants involved with both a home and community garden were equally likely as those working only in a home garden to be “concerned” or “very concerned” (55%). About 9.0% of all respondents said that they were “not concerned at all” about HMM soil contaminants. 

Participants with children and participants with lower incomes were also more likely to believe that HMM posed a very high risk to their health (Table 2 and Table 3). More than half of those with children believed HMM posed a very high risk to their health. In general, as average annual household income increased, the level of concern with eating produce in the garden decreased. For instance, 29% of respondents with average annual incomes of $0–$49,999 said that they had concerns about eating produce grown in their gardens, as opposed to 20% of respondents with average annual incomes of $50,000-$89,999, and 13% of respondents with average annual incomes of $90,000 or more (Table 3). 

In terms of race, white/Caucasian respondents were likely to indicate that they were aware of what slag was (Table 4). 44% of White/Caucasian respondents could accurately describe slag as an industrial byproduct with high levels of HMM, in comparison to 27% of black/African American respondents (Table 4). 16% of all participants answered that they were aware of what slag was, but could not accurately describe it as an industrial byproduct containing potentially harmful HMM contaminants. Respondents with different household incomes also varied in their knowledge of slag and ability to accurately describe characteristics and sources of slag (Table 4). 

The majority of participants were aware of and able to accurately describe the potential health effects of contaminants, such as lead and arsenic, regardless of income levels. Overall, 55% of participants indicated that they were aware of these health effects. White/Caucasian participants were again more likely to state that they were aware of these health effects, with 64% of white/Caucasian participants answering that they were aware, as opposed to 27% of black/African American participants answering that they were aware. 

One survey respondent noted that she was concerned about the health effects of chemicals and pesticides much more than soil contaminants. Another participant expressed concern that she, along with other gardeners in her community, lacked appropriate knowledge to detect the health effects of HMM. She also addressed the idea that community members may not want to have their soils tested for HMM or further their awareness of this topic:

My sense is that people [in my community garden] maybe suspect that there could possibly be some potential source of contamination. But they wouldn’t even really know what those contaminants could even be. Like, what [contaminants] might be likely considering the location?... I certainly don’t know how I would recognize the effects, you know, if [HMM] were having some effect on what I was growing or even my own health. I wouldn’t know what [effects] to look for, and I would suspect that’s probably true among the community as a whole... And it might be a bit of denial or avoidance [that causes this unawareness]. I actually recently had a conversation with a gardener about this, and she was like “You know, I don’t know that I want to know. This [plot of land] is what I have to work with, so I have to just continue to do what I’m doing either way.”

Similarly, another participant described:

Nobody [in my garden] has ever asked about [HMM soil contaminants]. Nobody’s ever questioned anything. We don’t know if it’s contaminated, we don’t know what do about it, we don’t ever think about it. But I think everyone would be interested [in learning more], especially because our garden is located on the site of a former city dump… But if I found out my soil was contaminated, I would of course want to remove all the contaminants from the soil. But I don’t know who I would even go to for that.

In many instances, participants who were more involved with gardening activities, such as those who participated in both home and community gardens, had increased knowledge of and expressed increased concern for the health effects of HMM contaminants. In fact, 73% of respondents engaged with both community and home gardens stated that they were aware of the potential health effects of slag, lead, and/or arsenic, in comparison to 40% of those involved only with community gardens and 55% of those involved only with home gardens (Table 5). One study participant was a leader in her gardening organization and had more than five years of experience with gardening. She said that because of her involved role in the garden organization, she was knowledgeable about the site history of her garden, which made her concerned about the potential HMM soil contamination. For instance, she described that one garden used to be “an industrial dumping ground” and “sits right next to a busy road in the city.” 

### 3.4. Sources of Exposure to Contaminants 

One participant said that he was highly concerned about “soil contamination living in an urban, post-industrial city and gardening next to an old home [built in 1950].” However, he added that the only strategy he was aware of to protect his health from these contaminants was to peel root vegetables before consumption. He stated:

Everyone in an urban environment signs up for added health hazards, from our soil, air, and water to vehicular accidents and high crime in the city. I believe the overall benefits of home and community gardening outweigh the possible soil contaminants issue. But I want to learn [to] make better decisions… There is a lot of information [regarding resources] online, but it is hard to know what sources to trust.

This participant described that he was highly concerned with soil contamination, but did not know enough about sources of exposure to take preventative measures. This opinion and gap in knowledge seemed to be quite common among survey respondents as well. In fact, 59% of all individuals who answered the survey said that they did not know of any possible sources of exposure to soil contaminants. Among the respondents who said that they were aware of possible sources of exposure to soil contaminants, few were able to list more than one potential source. 

**Figure 3:**
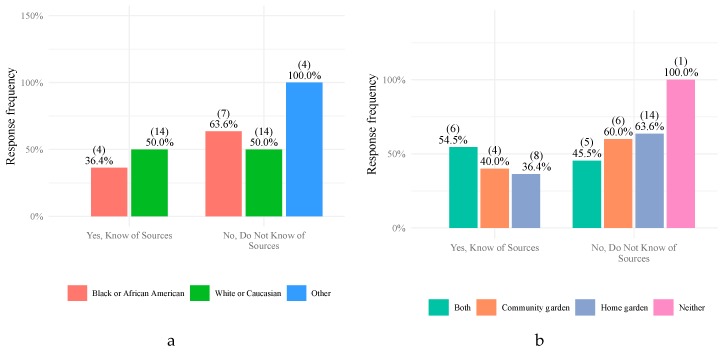
Knowledge of participants regarding potential sources of exposure to soil contaminants based on race (**a**) and type of garden (**b**).

Again, there were differences by race and types of gardens participants involved regarding their knowledge of potential soil HMM sources. Half of all white/Caucasian participants said that they were aware of sources of exposure, while only 36% of black/African American participants were aware (Figure 3a). Fifty-five percent of participants involved with both home and community gardens were knowledgeable about one or more sources, while 40% of participants involved with community gardens, and 36% of participants involved with home gardens were aware (Figure 3b). An increase in average annual household income was also correlated with a decline in a participant’s likelihood of knowing any sources of exposure. This result may be due to higher-income individuals believing that soil contamination was not a problem that personally affects them, and thus, doing less individual research and education on the subject. However, additional studies are needed to study this possible reasoning and validate this hypothesis.

### 3.5. Access to Soil Testing and Remediation Resources 

**Figure 4:**
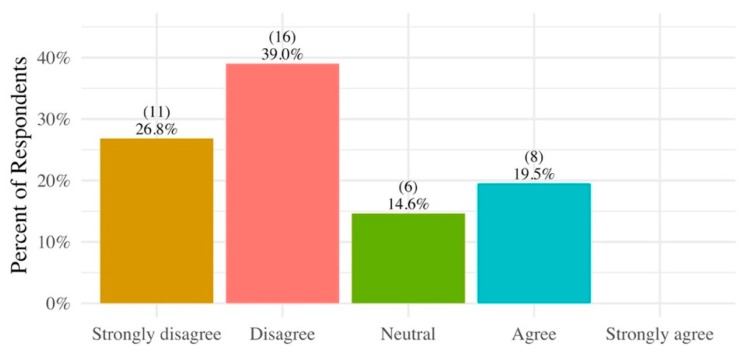
Knowledge of participants regarding methods for remediating HMM soil contaminants and/or slag.

A goal of the study was to identify communities in need of additional resources for soil remediation and to provide outreach tools and educational opportunities, such as the ASF event. Overall, participants indicated that they were unaware of methods for remediating HMM soil contaminants and/or slag, regardless of income. When asked whether they were aware of ways to get rid of HMM soil contaminants and/or slag, 66% did not know (disagreed or strongly disagreed) and only 20% knew (agreed or strongly agreed) (Figure 4). Some participants suggested that they were aware that phytoremediation was a possibility, but they added that they did not know enough about specific seeds or methods needed to do this or where they could find more information. Other remediation techniques, such as raised beds, were infrequently described. 

One survey respondent noted that she was unaware of any soil testing/remediation resources other than private soil testing businesses. During a follow-up interview, this participant suggested that if she did want her soil to be tested, it would be inconvenient to do so:

I’ve thought about having my soil tested by [an] extension office. It’s not that I can’t get there, but you know, it’s just out of the way. It’s inconvenient, and it’s never on my mind. It’s just out of my normal range of doing things... The easiest thing would probably be if I could take a soil sample and just send it somewhere- if I could just stick [the sample] in a vile and put it in the mail and the results could come back to me. So I think for anybody, it’s just the time and inconvenience factor [that prevents us from having our soil tested.]

Alongside convenience, another participant described transportation as a limiting factor for having her soil tested. In a follow-up interview, a third participant stated that in the context of her garden, she did not have the budget necessary to remediate HMM in her soil. Instead, when the presence of HMM was detected in her soils, she and other garden participants designated the site as a “non-edible space” where they discontinued growing produce. She added that she was glad these non-edible spaces were not large because she was unsure how she would remediate the soil if it had taken up a larger portion of the garden space. She stated:

“Remediation would not only take money, but also time, and I don’t have that kind of time… We [members of the community garden] have actually had our soil tested before, and instead of removing the heavy metals, we would just designate that site as a non-edible space… Luckily it wasn’t really a big portion of the garden.”

The same participant added that written and/or printed educational materials should be distributed to gardeners. She acknowledged that although in-person classes and hands-on workshops would be more informative, this would be too difficult for her gardening community to coordinate among members and to schedule. Other participants also cited the importance of physical and digital references for gardeners, as well as an expert whom they could contact to ask specific questions about HMM soil contaminants. Additional data from the survey’s findings can be found in Appendix C *and* Appendix D.

## 4. Discussion

### 4.1. Main Findings 

Many participants seemed to express some concerns with the possibility of HMM contaminants, especially when describing the adverse health effects. Despite this, nearly 80% of participants were unconcerned with eating and buying produce grown in urban gardens. Interviews revealed that many respondents had never had their soil tested, even those who lived in former industrial sites. For some, this was a result of never thinking that soil testing was necessary, and for others, this was a matter of convenience. Given these findings, it is possible that while participants are aware of the health effects of HMM, they do not think that the amount of contamination is high enough to pose a significant risk to their health. Even though they have not had their soil samples tested to prove that it is safe, they did not believe the level of contamination would have a significant impact on them. Improved access to soil testing through cheaper and closer facilities and/or programs may help to better connect gardeners to the quality and safety levels of their soil on a more individualized level. 

It is also worthwhile to recognize that the majority of participants were unaware of possible sources of exposure to soil contaminants. For example, participants indicated varied knowledge of slag as a potential source of soil contamination. Local extension offices and community organizations should consider educating gardeners about possible sources of exposure. It would also be beneficial to inform gardeners of personal actions that they can take to reduce exposure, such as thoroughly washing produce, keeping garden plots far away from industrial sites and busy roads, and leaving garden tools outside of the home. Such actions can reduce harmful exposures, regardless of the awareness of whether contaminants exist in high concentrations. 

These findings closely support previous studies conducted. For instance, Kim et al. (2014) [12] identified low levels of concern and knowledge about HMM contaminants and a lack of confidence in the ability to reduce exposure and remediate soil. Both this study and Kim et al. (2014) [12] found that lead was the most frequently described contaminant because respondents infrequently mentioned other trace metals, such as arsenic. In both studies, the lack of confidence in listing potential soil remediation practices was particularly noticeable. Some interviewees in this study mentioned that they refrain from growing produce in a certain area if they find out the area is contaminated, and Kim et al. (2014) [12] likewise found that 50% of respondents view this as an adequate form of remediation. Both studies found that urban gardeners generally lacked knowledge regarding other remedial practices, such as phytoremediation. Unlike Kim et al. (2014) [12], however, this study found that respondents generally preferred indirect education tools, such as handouts and online information hubs, rather than interactive, in-person outreach. Additionally, the findings of this study closely resemble those of Harms et al. (2013) [28], who found that urban gardeners had minimal confidence in their knowledge of remedial resources. 

Particular attention should be paid to differences in findings between racial groups throughout this study. Some risk assessments of different racial groups were negligible. For instance, black/African American respondents were only minimally more concerned about the health effects of HMM in soil. The difference in responses between black/African American respondents and white/Caucasian respondents in this area was not large enough to draw a valid conclusion. This finding contrasts Wong et al. (2018) [46], suggesting that black/African American individuals are significantly more concerned with soil contamination. This contrast may be due to the location of the study or the level of education and knowledge among participants. 

However, in some cases, differences in survey results from various ethnic groups were more noticeable. White/Caucasian participants were much more likely to answer that they were aware of the health effects of HMM. This information is important to determine which racial groups and communities in metro-Atlanta should be targeted more for intervention strategies. Based on these results, black/African American communities may be more likely to benefit significantly from additional outreach efforts, such as classes, events, and educational materials regarding the health effects of soil contaminants and remediation efforts. 

This study also found that home gardeners tended to be much more aware of the potential health hazards of HMM in soil, compared to community gardeners. This finding is supported by the fact that home gardeners were much more likely to cite health as a significant benefit of engaging in gardening than community gardeners were. Since home gardeners were more likely to view improved health as a benefit, they may have been more likely to educate themselves on the health outcomes related to gardening. Future studies should seek to understand better why home gardeners are more likely than community gardeners to view health as a benefit. 

Community gardeners, more so than home gardeners, therefore, may also greatly benefit from additional outreach and education efforts. Differences in awareness between the community and home gardeners regarding sources of exposure and remediation methods were minimal. However, home gardeners appeared to have a significantly greater understanding of the adverse health effects of HMM. This outcome was surprising, especially because community gardeners frequently cited education and the sharing of knowledge and information as perceived benefits of working and/or volunteering in community gardens. This difference in knowledge could potentially be attributed to home gardeners engaging in more independent research on the subject rather than relying on the knowledge of their neighbors and community members. For home gardeners working with HWG, this may be due to their close interaction with community garden leaders who provide information about health, the environment, and gardening. Future studies could determine whether this is true through surveys and focus groups with community members. High-income participants were less likely to believe that HMM was a very high risk to their health. This result may be because high-income participants did not live in areas with high pollution, but further research is also needed to determine whether this is true. 

During interviews, participants brought up factors that may be important to address to improve awareness of this topic. For example, one participant addressed the fact that many gardeners in her community were reluctant to seek soil testing because they would not be able to remediate their soil or find other, uncontaminated plots of land to garden. This suggested that more focus should be placed on educating individuals about how they can easily access low-cost remediation resources. If individuals understand that remediation efforts are feasible options for them, they may be more inclined to test their soil for contaminants. Findings from Hunter (2019) [47] suggest that resources and training are important factors that influence the likelihood of community gardeners shifting their behavior to create healthier soil. Hunter et al. (2019) [48] also found that individuals would like to have more access to HMM soil testing, but were unable to due to lack of time and ease in doing so. 

The results from this study suggest a potential need to communicate and educate health exposures, soil testing, and remediation methods related to HMM better. Citizen science programs such as VegeSafe have provided free soil metal screening to communities, allowing researchers to gather data on HMM soil exposures, which they then report to community members. The program also advises community members on solutions if soils contain high levels of HMM [49]. Tools such as this one may benefit urban gardeners. 

The event “Getting Dirty: Exploring Soil on Atlanta Farms” at the ASF proved to be a successful outreach event that attracted 250 residents of metro-Atlanta, as well as individuals interested in beginning gardening. This outreach event, which provided a combination of both hands-on activities such as soil testing, as well as more informal opportunities to hand out educational flyers, allowed over 50 Atlanta residents to have their soil tested. Results from soil testing led to an Environmental Protection Agency (EPA) investigation and later excavation of HMM contaminated soil. This study provides information that supports the need for further outreach efforts. It also suggests black/African American communities should be targeted, as well as identifying which outreach methods should be used to most effectively improve awareness of HMM. The study also indicates that resources for soil testing and remediation should be more widely publicized and more accessible to urban gardeners. 

### 4.2. Concerns and Limitations 

Recruiting a large group of participants for the study was a challenge, and the sample size for the study was relatively small. This limited the extent to which these results can be generalized to other urban areas and to residents of Atlanta who did not participate in the study. Given the relatively small sample size, it was difficult to infer statistical significance. While this study can help to provide a broader understanding of community gardeners’ awareness of HMM, future studies should seek to gather a larger sample size. Differences in demographic characteristics between study participants and all residents of Atlanta may limit the generalizability of these findings to the population that gardens in Atlanta. According to the U.S. Census, 40.3% of Atlanta residents were white, and 51.8% were black or African American in 2018 [50]. These percentages are different than the demographic data found in our study population (61% white, 26% African American). This result may be because the demographic data of Atlanta gardeners is different from the demographic data of all Atlanta residents. The data from Hunter (2019) [47], which focused on garden leaders in Atlanta, had similar racial profile as ours (69% white, 27% African American), which supports this argument. 

The participants included in the study may have also skewed the data. Researchers attempted to contact a wide and diverse range of respondents through the American Community Gardening Association’s database and by collecting surveys at the ASF event, which was open and advertised to all Atlanta-area gardeners. However, many of the gardeners may still have been associated with HWG, which could have limited the ability to accurately generalize results to communities and residents outside of this organization with confidence. Additionally, participants recruited at the ASF or through HWG may have already received some information about HMM exposure from ASF events or HWG meetings. 

Since most respondents were either black/African American or white/Caucasian, it was especially difficult to compare data across races. For this reason, we were unable to draw valid conclusions about American Indian/Alaskan Native, Asian/Asian American, and Hispanic, Latino participants. Instead, we compared data across demographic factors that were more varied, such as average annual household income and type of garden participated. Additionally, it was possible that the knowledge of HMM soil exposure was correlated with the decision to participate in a garden. Under these circumstances, individuals with concerns about HMM exposure may have chosen not to participate in gardening and would not have been represented in this sample. 

A third possible limitation of this study is the non-response error. Given that gardeners provided with the survey were not required to complete the survey, some potential participants may have chosen not to participate in the study due to time constraints or disinterest. Some may also have been discouraged from completing the entirety of the survey due to its length. Future studies should minimize this error by attempting to recruit a larger number of respondents from the sample to obtain a normal distribution. 

### 4.3. Opportunities for Future Studies 

There are many opportunities for future studies related to measuring community awareness of HMM and determining best practices to improve awareness in communities in the future. Other social science methods, such as focus groups, can be used to continue to analyze community awareness of soil contaminants. A greater number of studies regarding this topic will help to validate the data by reaching a broader sample size and a wider variety of participants. 

It is important for future research to compare the information gained through this study with the measured levels of HMM in the soil of various Atlanta communities. This study did not gather information on whether respondents had their soil tested for HMM contaminants before, and what the results of the tests were. Researchers could distribute surveys to fill this gap in information, and they could also sample soils in metro-Atlanta gardens to determine if any areas with low levels of knowledge regarding soil contaminants had high levels of contamination. This research is important to effectively target outreach efforts to vulnerable populations. 

This study’s scope was limited to the metro-Atlanta area. Therefore, it is also important that follow-up studies be conducted outside of Atlanta. Other urban areas may have different levels of HMM, and thus, may have greater community awareness of these contaminants. Additionally, interventions may differ between cities. Future research should be conducted in previously unstudied areas to determine whether the results of this study were specific only to Atlanta. Additionally, it would be interesting to study related topics, such as gardeners’ cost threshold to have their soil tested or how far they would travel to do so. 

Given that urban agriculture has many benefits and is growing more popular, it is important to understand better how to improve the safety of gardeners. Additional studies can help to understand which areas gardeners lack knowledge concerning HMM. This information can then be used, especially in conjunction with outreach efforts, to inform community organizations on how to better educate gardeners and policy-makers on how they can make urban agriculture sites safe and soil testing more accessible, as well as individuals on how they can continue to garden safely. Such studies are especially significant to target the most vulnerable populations and efficiently improve the safety and well-being of urban gardeners. 

## 5. Conclusions

A combination of surveys and interviews with gardeners and farmers in metro-Atlanta provided an opportunity to understand better the risks they perceived concerning HMM contaminants and remediation resources. Although many participants were aware and concerned about the potential health effects of HMM contaminants, they believed that they were not personally at risk. Many respondents lacked knowledge of sources of HMM exposures and methods for mediation. Few gardeners had their soil tested for HMM contaminants before this study. Home gardeners and white/Caucasian participants tended to be most informed about HMM contamination and remediation. Results from this study suggest a need for further community education and outreach on this subject. 

## Figures and Tables

**Table 1:** Concern of participants with potential health effects of HMM contaminants in soil by race (top) and by types of gardens (bottom).

Race	Not Concerned at All	Not very Concerned	Neutral	Concerned	Very Concerned
Black or African American	9.1%	18%	27%	27%	18%
White or Caucasian	11%	29%	7.1%	46%	7.1%
**Type of Garden Both**	9.1%	27%	9.1%	36%	18%
Community garden	10%	30%	20%	30%	10%
Home garden	9.1%	18%	18%	50%	4.6%
Neither	0%	0%	0%	0%	1.0%

Total question respondents: 44.

**Table 2:** Perceived risk of HMM soil contaminants on health by participants with and without children.

Children or No Children	Very Low Risk	Low Risk	Neutral	High Risk	Very high Risk
Children	0%	6.7%	17%	23%	53%
No Children	0%	14%	29%	43%	14%

Total question respondents: 44.

**Table 3:** The concern of participants with eating or buying produce grown in gardens by income level.

Income Bracket	Yes, Have Been Concerned	No, Have Not Been Concerned
$0–$49,999	29%	71%
$50,000–$89,999	20%	80%
$90,000 or more	13%	87%

Total question respondents: 44.

**Table 4:** The ability of participants to accurately describe slag by race (top) and by income levels (bottom).

Race	Yes, Know What Slag Is	No, Do Not Know What Slag Is
Black or African American	27%	73%
White or Caucasian	44%	56%
**Income Bracket**		
$0–$49,999	38%	63%
$50,000–$89,999	30%	70%
$90,000 or more	38%	63%

Total question respondents: 43.

**Table 5:** Knowledge of participants regarding potential health effects of slag, lead, and/or arsenic in soil based on the type of garden they participated in.

Type of Garden	Yes, Know of Health Effects	No, Do Not Know of Health Effects
Both	73%	27%
Community garden	40%	60%
Home garden	55%	46%

Total question respondents: 44.

**Figure A1:**
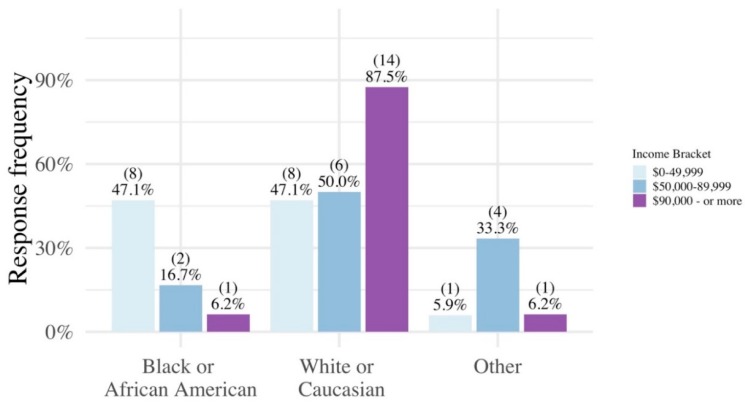
Income vs. Race of Participants. *Total question respondents: 51.*

**Figure A2:**
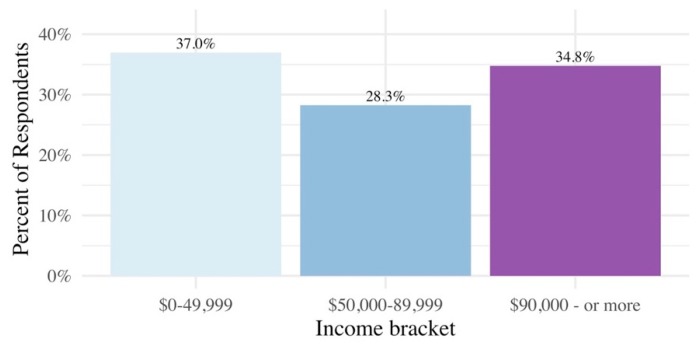
Average annual household income among participants. Total question respondents: 51.

**Figure A3:**
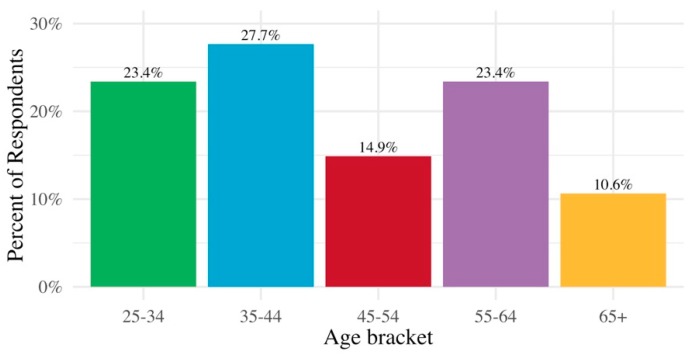
Age of participants. Total question respondents: 51.

**Figure A4:**
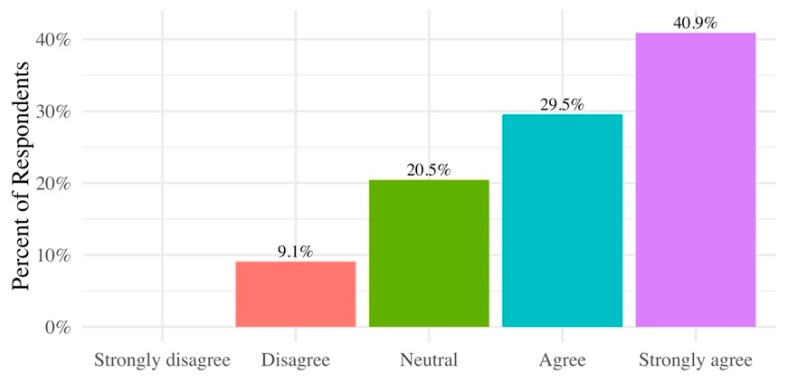
Participants’ perceived risk of heavy metal soil contaminants on health. Total question respondents: 44.

**Figure A5:**
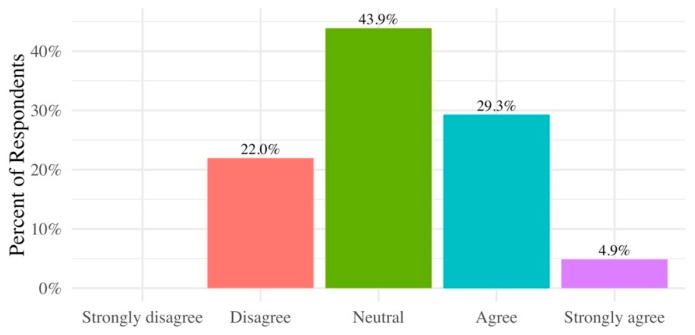
Knowledge of participants regarding potential soil remediation methods. Total question respondents: 41.

**Figure A6:**
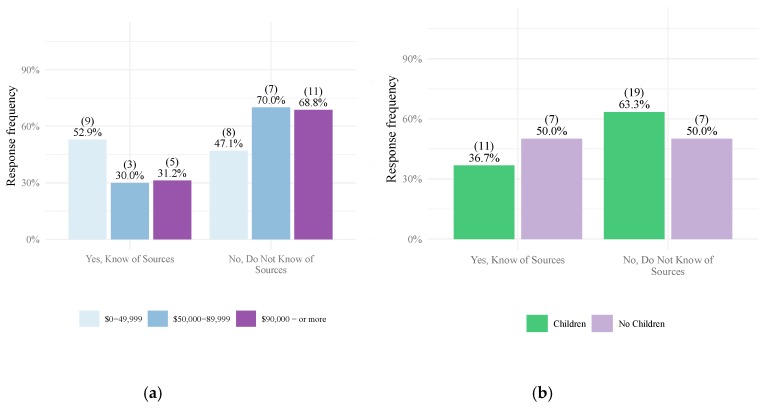
Knowledge of participants regarding potential sources of exposure to soil contaminants based on income (**a**) and the existence of children (**b**). Total question respondents: 41.

**Figure A7:**
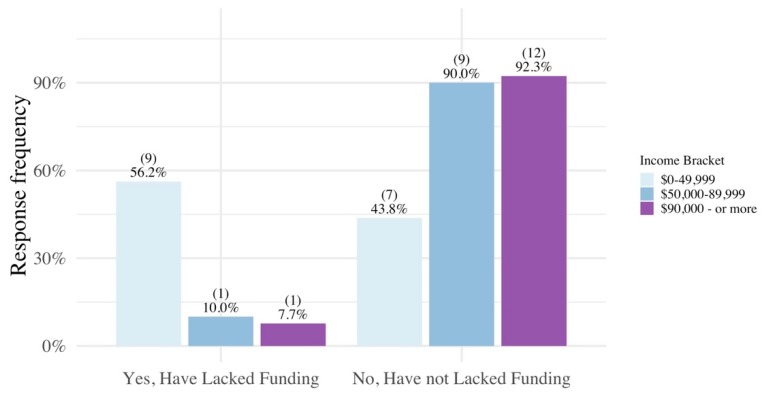
Respondents’ lack of funding to remediate heavy metal soil contaminants and/or slag by income levels. Total question respondents: 40.

**Figure A8:**
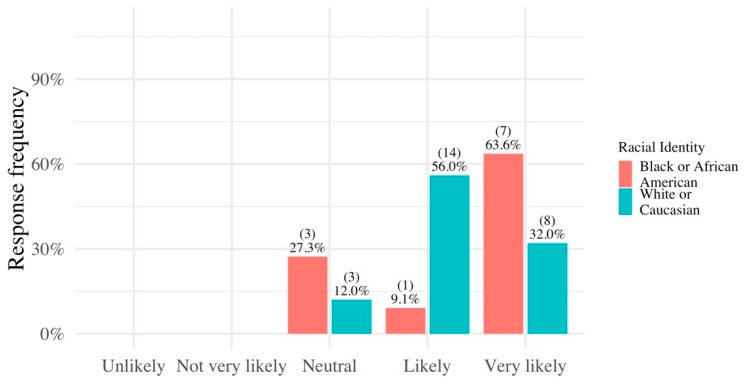
Likelihood of participants taking action to remove heavy metal soil contaminants if made aware of them by racial identity. Total question respondents: 41.

## Appendix A—Survey Questions

Directions: Please circle your answer and write your explanation if necessary.

1. Consent Form Signature
2. What is your race? Please circle all that apply.
  - White or Caucasian
  - Black or African-American
  - Hispanic or Latino
  - American Indian or Alaskan Native
  - Asian or Asian America
  - Native Hawaiian or other Pacific Islander
  - Other (please specify): __________________
3. What is your annual household income (how much total combined money did all members of your household earn in 2018)?
  - $0–$9,999
  - $10,000–$29,999
  - $30,000–$49,999
  - $50,000–$69,999
  - $70,000–$89,999
  - $90,000–or more
4. What is your age?
  - 18–24
  - 25–34
  - 35–44
  - 45–54
  - 55–64
  - 65+
5. What is your gender?
  - Male
  - Female
  - Other
6. Which neighborhood do you live in? ____________________________________________
  How long have you lived in your neighborhood?
  - Less than 1 year
  - 1–2 years
  - 3–5 years
  - 6–7 years
  - 8–10 years
  - Longer than 10 years
7. Do you have any children?
  - Yes
  - No
8. If you answered yes to #7, how old are your children? If you answered no, please write N/A. _______________________________
9. How many people live in your household? _______________________________________
10. 10. What type of garden do you participate in? Please select all that apply:
  - Home garden
  - Community garden (please specify which one): _________________
11. If you participate in a home garden, what do you see as the benefits of participating in a home garden? _________________________________________________________________________________
12. If you participate in a community garden, what do you see as the benefits of participating in a community garden?
  _________________________________________________________________________________
13. In what capacity do you participate in your garden(s) (i.e., tend to the garden, help with weed clean-up, eat produce, etc.)?
  _________________________________________________________________________________
14. How many days a week do you participate in your garden(s)?
  - 1 day a week
  - 2–3 days a week
  - 4–5 days a week
  - 6–7 days a week
15. What is the primary purpose of the food you grow in your gardens? Please select all that apply.
  - Personal consumption
  - Family consumption
  - To sell to others
  - Other: ________________
16. Do you eat produce grown in your garden(s)?
  - Yes
  - No
17. If you answered yes to #16, please describe. For example, which foods do you eat? How often? How much? Do you ever sell the produce to other community members or feed it to your family? _________________________________________________________________________________
18. During your time living here, have you had any concerns (for you and/or your family) about eating and buying produce grown in your gardens?
  - Yes
  - No
19. If you answered yes to #18, please describe:
  _________________________________________________________________________________
20. During your time living here, have you had any concerns (for you and/or your family) about the health effects related to how long or much you are exposed to soil? Please describe:
  _________________________________________________________________________________
21. Do you know what slag is?
  - Yes
  - No
22. If you answered yes to #21, please describe: _________________________________________________________________________________
23. Do you know about any of the potential health effects of slag, lead, or arsenic in soil?
  - Yes
  - No
24. If you answered yes to #23, please explain:
  _________________________________________________________________________________
25. How concerned are you about the potential health effects of heavy metal contaminants (lead, arsenic, chromium, etc.) in soil?
  - Very concerned (I think about it every day)
  - Concerned (I don’t think about it every once in a while when I’m gardening)
  - Neutral
  - Not very concerned (I know about the health effects, but am not worried about them)
  - Not concerned at all (I have never thought about the health effects and/or am unaware of them)
26. Do you know about any of potential sources of exposure to soil contaminants?
  - Yes
  - No
27. If you answered yes to #26, please explain:
  _________________________________________________________________________________
28. Do you think heavy metals in soil pose a significant risk to your health?
  - Strongly agree
  - Agree
  - Neutral
  - Disagree
  - Strongly disagree
29. Please explain your answer to #28: _________________________________________________________________________________
30. Do you know what resources you have to learn more about heavy metal contaminants in soil?
  - Strongly agree
  - Agree
  - Neutral
  - Disagree
  - Strongly disagree
31. Please explain your answer to #30 and if applicable, list any resources you are aware of:
  _________________________________________________________________________________
32. Do you know about ways to get rid of heavy metal soil contaminants and/or slag?
  - Strongly agree
  - Agree
  - Neutral
  - Disagree
  - Strongly disagree
33. Please explain your answer to #32, and if applicable, list any methods you are aware of:
  _________________________________________________________________________________
34. Have you ever wanted to get rid of heavy metal soil contaminants and/or slag, but did not have the resources and/or funding to do so?
  - Yes
  - No
35. Please explain your answer to #34:
  _________________________________________________________________________________
36. How likely would you be to take action to remove heavy metal soil contaminants from soil if you were made aware of them?
  - Very likely
  - Likely
  - Neutral
  - Not likely
  - Not likely at all
37. Would you be available and interested in an additional, in-person interview to further discuss this topic? The interview would last about 20–30 minutes. It would allow you to provide more detailed information about your answers to this survey and would provide a chance to learn more about heavy metal contaminants and resources you can access to learn more. If so, please provide your name and best method of contact below. (This information will ONLY be used to contact you for an in-person interview.)
  Name: __________________________ .
  Email: __________________________ .

## Appendix B—Demographic Tables and Graphs

**Table A1:** Demographic data of participants. Race of participants.

Race	Number of Respondents	Percent of Respondents
American Indian or Alaskan Native	1	2.2%
Asian or Asian American	3	6.5%
Black or African American	12	26%
Hispanic or Latino	2	4.3%
White or Caucasian	28	61%
Preferred Not to Answer	5	N/A

**Table A2:** Household income of participants.

Income Bracket	Number of Respondents	Percent of Respondents
$0–$49,999	17	37%
$50,000–$89,999	13	28%
$90,000 or more	16	35%
Preferred Not to Answer	5	N/A

**Table A3:** Type of garden used by participants.

Type of Garden	Number of Respondents	Percent of Respondents
Both	11	22%
Community garden	12	23%
Home garden	23	45%

Notes: 9.8% of respondents were not actively gardening, but had previously gardened or were preparing to start a garden.

**Table A4:** Gender of participants.

Gender	Number of Respondents	Percent of Respondents
Male	14	32%
Female	32	68%

**Table A5:** Frequency of participants having children.

Children vs. No Children	Number of Respondents	Percent of Respondents
Children	31	66%
No Children	16	34%
Preferred Not to Answer	4	N/A

Note: Percentages calculated with only valid responses (i.e., N/A responses not included in denominator).

## Appendix C—Analytical Tables

**Table A6:** Frequency of participants eating the produce grown in their gardens.

Race	Yes, Eat Produce	No, Do Not Eat Produce
Black or African American	91%	9.10%
White or Caucasian	93%	7.10%
**Income Bracket**		
$0–$49,999	94%	5.90%
$50,000–$89,999	90%	10%
$90,000 or more	94%	6.30%

Total question respondents: 44.

**Table A7:** Participants’ level of concern regarding eating and buying produce grown in their gardens.

Race	Yes, Have Been Concerned	No, Have Not Been Concerned
Black or African American	18%	82%
White or Caucasian	25%	75%
**Children vs. No Children**		
Children	13%	87%
No Children	36%	64%
**Type of Garden**		
Both	18%	82%
Community garden	20%	80%
Home garden	23%	77%

Total question respondents: 44.

**Table A8:** Participants’ knowledge regarding potential health effects of slag, lead, and/or arsenic in soil.

Race	Yes, Do Know of Health Effects	No, Do Not Know of Health Effects
Black or African American	27%	73%
White or Caucasian	64%	36%
**Income Bracket**		
$0–$49,999	47%	53%
$50,000–$89,999	40%	60%
$90,000 or more	50%	50%
**Children vs. No Children**		
Children	53%	47%
No Children	57%	43%

Total question respondents: 44.

**Table A9:** Participants’ concern regarding potential health effects of heavy metal contaminants in soil.

Participant Demographic	Not Concerned at All	Not Very Concerned	Neutral	Concerned	Very Concerned
$0–$49,999	5.9%	18%	12%	41%	24%
$50,000–$89,999	20%	20%	20%	40%	0%
$90,000 or more	6.3%	25%	19%	44%	6.3%
Children	6.7%	23%	17%	43%	10%
No Children	14%	21%	14%	36%	14%

Total question respondents: 44.

**Table A10:** Participants’ perceived risk of heavy metal soil contaminants on health.

Race	Very low risk	Low risk	Neutral	High risk	Very high risk
Black or African American	0%	9.10%	9.10%	27%	55%
White or Caucasian	0%	11%	25%	36%	29%
**Income Bracket**					
$0–$49,999	0%	12%	18%	29%	41%
$50,000–$89,999	0%	20%	30%	20%	30%
$90,000 or more	0%	20%	19%	31%	50%
**Type of Garden**					
Both	0%	18%	18%	46%	18%
Community garden	0%	0%	40%	30%	30%
Home garden	0%	9.10%	14%	23%	55%

Total question respondents: 44.

**Table A11:** Participants’ knowledge of soil remediation methods.

Race	Very weak knowledge	Weak knowledge	Neutral	Strong knowledge	Very strong Knowledge
Black or African American	27%	46%	18%	9.10%	0%
White or Caucasian	24%	32%	16%	28%	0%
**Income Bracket**					
$0–$49,999	18%	35%	18%	29%	0%
$50,000–$89,999	10%	60%	20%	10%	0%
$90,000 or more	46%	31%	7.70%	15%	0%
**Type of Garden**					
Both	27%	27%	18%	27%	0%
Community garden	13%	38%	25%	25%	0%
Home garden	29%	48%	9.50%	14%	0%

Total question respondents: 41.

**Table A12:** Knowledge of participants regarding resources to learn about soil remediation.

Race	Very low knowledge	Low knowledge	Neutral	High knowledge	Very high knowledge
Black or African American	0%	18%	55%	18%	9.10%
White or Caucasian	0%	24%	36%	36%	4.00%
**Income Bracket**					
$0–$49,999	0%	18%	47%	29%	5.90%
$50,000–$89,999	0%	30%	40%	20%	10%
$90,000 or more	0%	23%	46%	31%	0%
**Type of Garden**					
Both	0%	18%	46%	36%	0%
Community garden	0%	38%	38%	25%	0%
Home garden	0%	19%	48%	24%	9.50%

Total question respondents: 41.

**Table A13:** Lack of funding to remediate heavy metal soil contaminants and/or slag.

Race	Yes, Have Lacked Funding	No, Have Not Lacked Funding
Black or African American	30%	70%
White or Caucasian	32%	68%
**Income Bracket**		
$0–$49,999	56%	44%
$50,000–$89,999	10%	90%
$90,000 or more	7.70%	92%
**Type of Garden**		
Both	46%	55%
Community garden	29%	71%
Home garden	19%	81%

Total question respondents: 40.

**Table A14:** Likelihood of participants taking action to remove heavy metal soil contaminants if made aware of them.

Race	Not very likely	Unlikely	Neutral	Likely	Very likely
Black or African American	0%	0%	27%	9.10%	64%
White or Caucasian	0%	0%	12%	56%	32%
**Income Bracket**					
**$0–$49,999**	**0%**	**0%**	**18%**	**35%**	**47%**
$50,000–$89,999	0%	0%	10%	50%	40%
$90,000 or more	0%	0%	23%	31%	46%
**Children vs. No Children**					
Children	0%	0%	19%	33%	48%
No Children	0%	0%	14%	43%	43%
**Type of Garden**					
Both	0%	0%	0%	46%	55%
Community garden	0%	0%	25%	38%	38%
Home garden	0%	0%	24%	33%	43%

Total question respondents: 41.

## Appendix D—Analytical Graphs

All figures are graphed as response frequency (y-axis) vs. answer to survey question (x-axis).
